# Comparative analysis of CRP and PCT as biomarkers for early diagnosis of pediatric septic arthritis: superior performance of CRP

**DOI:** 10.3389/fped.2025.1582978

**Published:** 2025-10-14

**Authors:** Wenchen Xu, Piaoxue Yu, Xiangxuan Wang, Kainan Lin, Hui Chen

**Affiliations:** ^1^ Department of Orthopedics, Fujian Children’s Hospital (Fujian Branch of Shanghai Children’s Medical Center), College of Clinical Medicine for Obstetrics & Gynecology and Pediatrics, Fujian Medical University, Fuzhou, China; ^2^ Department of Ultrasonography, Fuzhou Second Hospital, Fuzhou, China

**Keywords:** septic arthritis (SA), early diagnosis, pediatric, C-reactive protein (CRP), procalcitonin (PCT), biomarkers, infection

## Abstract

**Background:**

Septic arthritis is a severe pediatric infection that affects joint health and requires early diagnosis to prevent complications. Traditional methods have limitations, and although serum PCT shows promise as a diagnostic marker, its efficacy remains controversial, warranting further studies.

**Objective:**

To investigate the value of C-reactive protein (CRP) and procalcitonin (PCT) in the early diagnosis of septic arthritis (SA) in pediatric patients and to compare their diagnostic efficiencies.

**Methods:**

This investigation utilized a retrospective cohort methodology to ascertain and compare serum procalcitonin (PCT) and C-reactive protein (CRP) concentrations among a pediatric population comprising 29 individuals diagnosed with septic arthritis and 25 with non-septic arthritis (NSA), all of whom were admitted to our institutional facility over a three-year period from 2019 to 2021.

**Results:**

CRP levels (>10 mg/L) were significantly higher in the septic arthritis group than in the non-septic group (26/29 vs. 3/25, *P* < 0.001), whereas PCT levels (>0.25 ng/ml) showed no significant difference (5/29 vs. 1/25, *P* = 0.385). ROC analysis revealed a high diagnostic performance for CRP (AUC 0.950, 95% CI 0.886–0.995, Youden index 88.6%) compared with PCT (AUC 0.574, 95% CI 0.417–0.731, Youden index 17.2%), indicating the superior sensitivity and specificity of CRP for early diagnosis of septic arthritis.

**Conclusion:**

Our findings substantiate the substantial superiority of CRP for the early detection and preliminary alert of pediatric septic arthritis. However, due to the small sample size, its significant advantage over procalcitonin (PCT) requires confirmation in larger studies.

## Introduction

Septic arthritis (SA) is an infectious disease that significantly affects children's growth and development, accounting for approximately 41% of pediatric orthopedic infectious diseases (1). While Septic arthritis can occur at any age, it commonly affects joints such as the hips, knees, ankles, and elbows in children (2). The cardinal clinical manifestations include erythema, swelling, warmth, and pain in the affected joints, frequently accompanied by restricted mobility. Epidemiologically, the incidence of pediatric septic arthritis in developed countries ranges from 2 to 10 cases per 100,000 children annually, whereas in developing nations, this figure may escalate to 20 cases per 100,000 children per year (3). Pediatric septic arthritis is an extremely serious disease that requires urgent treatment in pediatric orthopedics, even more urgent than some fractures and joint injuries. Delayed infectious joint disease may lead to severe joint destruction, osteomyelitis, and even limb function loss, with an incidence rate as high as 29% (4). Failure to promptly treat this condition may result in complications such as systemic infection and unequal limb development, which can significantly impede healthy childhood development (5). Hip joint infections are particularly high-risk, often resulting in femoral head necrosis or hip dislocation, necessitating surgical reconstruction (6). Consequently, early diagnosis and prompt therapeutic intervention are critical for effective disease management and the prevention of irreversible complications.

In the past, early diagnosis of infectious arthritis in children was based on the classic Kocher criteria (7). With advancements in rapid blood testing, conventional inflammatory markers such as the erythrocyte sedimentation rate (ESR) and C-reactive protein (CRP) levels have been adopted as early warning indicators for SA. Among these, CRP is currently one of the primary recommended laboratory tests, demonstrating substantial value in both SA diagnosis and therapeutic monitoring (8). CRP, synthesized by liver cells induced by tumor necrosis factor released by monocytes, generally within 24–48 h of onset and peaks (9). It is widely accepted that CRP is very sensitive to the early diagnosis of infectious diseases and can be used as an indicator to accurately reflect the severity of inflammation (10, 11). A related review by Mathews demonstrator's good utility in identifying septic arthritis (12).

With a high sensitivity (96%) and specificity (88%) for detecting conditions like sepsis and bacteremia (13), procalcitonin (PCT)—the precursor of calcitonin (CT)—has become a well-established biomarker for systemic bacterial infections. PCT and CRP, as acute-phase response proteins, differ in their production mechanisms and time course. In a typical physiological milieu, the parathyroid glands orchestrate the transformation of PCT to CT. In healthy individuals, PCT synthesis is suppressed by a physiological feedback mechanism. However, the presence of endotoxin disrupts this mechanism, inhibiting the conversion of PCT to calcitonin and culminating in the release of PCT fragments into the bloodstream, reaching detectable levels (14). Concomitantly, inflammatory cytokines induce the synthesis and secretion of procalcitonin (PCT) by hepatic macrophages and monocytes, as well as by lymphocytes and endocrine cells within the pulmonary and intestinal tissues, representing an additional substantial origin of PCT (15). With the increasing popularity of serum PCT in the study of bacterial infection-related diseases, some scholars have applied PCT to the diagnosis of septic arthritis and achieved certain results (16).

Nevertheless, contradictory evidence indicates that the clinical importance of PCT might not meet expectations, while its reliability for SA diagnosis remains a subject of debate (17, 18). The debate stems from the question of whether serum PCT, as a diagnostic marker reflecting systemic infection, remains highly sensitive when early joint infections do not spread throughout the body. To further examine this controversy, we designed a retrospective study to evaluate serum PCT and CRP levels in children with or without septic arthritis admitted to our hospital, aiming to compare the diagnostic efficacy of PCT and CRP for septic arthritis (SA).

## Patients and methods

This study was approved and monitored by the Ethics Committee of Fujian Children's Hospital, in accordance with the ethical guidelines of the 1964 Declaration of Helsinki. Owing to the retrospective design of this study, the Institutional Review Board granted a waiver of informed consent.

### Patients & methods

Between January 2019 and December 2021, 67 patients presenting with acute joint erythema, swelling, warmth, and pain were considered for this study. After applying the exclusion criteria, a final cohort of 54 children were enrolled in the study. The sample size was determined based on the following factors: the low incidence rate of septic arthritis in this region (approximately 10 cases per year); strict inclusion and exclusion criteria to ensure high diagnostic accuracy and consistency among the enrolled cases; limited research time; and the single-center experimental design.

### Diagnostic criteria for septic arthritis

The diagnosis of septic arthritis in our study was based on the gold standard of joint fluid culture, which is widely recognized in the medical community for its high specificity and reliability in identifying the causative pathogens (19). Our study adhered to this established criterion, utilizing joint fluid culture positivity as the definitive diagnostic benchmark to evaluate the comparative efficacy of PCT and CRP as early diagnostic biomarker. In this study, we included cases with positive synovial fluid cultures in the SA group, while cases with negative cultures were included in the NSA group.

### Inclusion and exclusion criteria

#### Inclusion criteria

1. Clinical Presentation: The patient presented with joint pain, swelling, and limited mobility, suggesting possible septic arthritis. 2. Age Range: Children aged between 0 and 18 years old. 3. Treatment History: No prior antibiotic treatment.

#### Exclusion criteria

1. Coexisting Severe Conditions: Presence of other serious diseases, including cardiac, hepatic, renal dysfunction, immunological disorders, oncological diseases, or chronic systemic inflammatory diseases. 2. Immunological Disorders: History of immunodeficiency or long-term use of immunosuppressive agents. 3. Treatment History: Any antibiotic treatment prior to enrollment or any surgical history within the last 3 months.

### Sterile joint fluid aspiration and culture protocol

#### Sampling standards

All joint aspirations were performed by experienced pediatric orthopedic surgeons. The procedure was conducted in an operating room under strict aseptic conditions, including full surgical scrub, sterile gown, gloves, mask, and cap, with the affected joint draped widely. An attempt was made to collect a minimum of 1–2 ml of synovial fluid. The fluid was immediately inoculated directly into blood culture bottles at the bedside.

#### Culture methods

1. Primary Culture: Joint fluid was inoculated into anaerobic blood culture bottles (Becton-Dickinson, Germany) or BactecPeds Plus/F blood culture bottles (Becton-Dickinson, Germany) and incubated in a Bactec 9050 automatic thermostat (Becton-Dickinson, Germany) for up to 5 days. 2. Secondary Culture: Any bottle flagged as positive samples was immediately subcultured onto specialized media: Aerobic bacteria: Blood agar plates; Anaerobic bacteria: Anaerobic culture media. Gram staining was performed on all fluid samples prior to culture.

#### Differentiation of CoNS pathogens from contaminants

To minimize misclassification, we applied strict criteria to differentiate CoNS as true pathogens from potential contaminants. A case of CoNS was considered a true pathogen causing bloodstream infection only if it met at least one of the following conditions: (1) the same CoNS species was isolated from two or more separate blood culture sets drawn from different sites; (2) the patient exhibited unequivocal clinical signs of septic arthritis consistent with the infection; or (3) the patient demonstrated a clear clinical and microbiological response (e.g., resolution of fever and leukocytosis, negative follow-up cultures) following the initiation of targeted anti-staphylococcal therapy. Cases not meeting these criteria were excluded from the final analysis as probable contaminants.

### Identification techniques

Gram and acid-fast staining were used for bacterial identification.

### Laboratory analyses and quality control

Serum CRP concentrations were quantified using a particle-enhanced immunoturbidimetric assay on a Roche Cobas® 8000 modular analyzer (Roche Diagnostics, Mannheim, Germany), according to the manufacturer's instructions. The assay's detection limit was 0.3 mg/L.

Serum PCT levels were measured using an electrochemiluminescence immunoassay (ECLIA) on a Roche Cobas® e601 analyzer (Roche Diagnostics, Mannheim, Germany). The functional assay sensitivity was 0.06 ng/ml.

The analyses were performed in a laboratory accredited by ISO 15189. To ensure analytical reliability, internal quality control (IQC) was rigorously implemented. For the CRP assay, IQC was conducted twice daily using commercial control materials. For the PCT assay, IQC was performed once daily. This protocol guarantees that the assays consistently operate within validated performance parameters.

The reference ranges for CRP and PCT were defined as 0–10 mg/L and 0–0.25 ng/ml, respectively, in accordance with minimal standards suggested by peers to optimize diagnostic sensitivity.

### Data collection

For each child, records of blood and synovial fluid sample collection were reviewed, confirming that both specimens were obtained concurrently at the time of initial presentation, prior to any therapeutic intervention. Retrospective data on synovial fluid culture results and serum CRP and PCT levels were then extracted from the medical records.

The timing of sample collection was meticulously recorded and defined as the interval from the onset of symptoms (e.g., fever, joint pain, swelling, or limping) to the time of blood draw for CRP and PCT measurement. For the purposes of this study, symptom onset was operationally defined as the earliest time at which symptoms were either observed by the guardian or self-reported by the patient. This information was extracted from patient medical records and is summarized in Table 1.

**Table 1:** Baseline characteristics of the participants.

Variate	Total (*n* = 54)	Septic arthritis (SA)		*P* value
		Y (*n* = 29)	*N* (*n* = 25)	
Age, M (IQR), y	4 (2–8)	4 (2–7)	4 (2–8)	0.948
Gender, *n* (%)				0.113
Female	24 (44.444)	10 (34.483)	14 (56.000)	
Male	30 (55.556)	19 (65.517)	11 (44.000)	
Diseased site, *n* (%)				0.010
Hip	26 (48.148)	9 (31.034)	17 (68.000)	
Knee	17 (31.481)	9 (31.034)	8 (32.000)	
Shoulder	4 (7.407)	4 (13.793)	0 (0.000)	
Ankle	3 (5.556)	3 (10.345)	0 (0.000)	
Sternoclavicular	2 (3.704)	2 (6.897)	0 (0.000)	
Elbow	2 (3.704)	2 (6.897)	0 (0.000)	
Time of sample collection, days, *n* (%)				0.751
≤2 days	11 (20.4)	5 (17.2)	6 (24.0)	
3–5 days	18 (33.3)	11 (37.9)	7 (28.0)	
6–7 days	9 (16.7)	5 (17.2)	4 (16.0)	
>7 days	16 (29.6)	8 (27.6)	8 (32.0)	

M, median of dataset; Q1, first quartile of dataset; Q3, third quartile of dataset.

The diagnostic performances of PCT and CRP were assessed independently and are presented as receiver operating characteristic (ROC) curves, areas under the curve (AUCs), and Youden indices.

### Statistical analysis

Continuous variables (CRP/PCT levels) are expressed as median [interquartile range, M (Q1–Q3)] to accommodate potential non-normal distributions. Categorical variables were summarized as frequencies and percentages. Statistical analyses were performed using SPSS software (version 26.0). The t-test was used to compare continuous variables between groups, while the chi-square test (Chi2) was used for categorical data. The diagnostic efficacy was evaluated using ROC analysis, including the calculation of the AUC. Statistical significance was set at *p* < 0.05.

## Results

### Comparison of baseline characteristics between the SA and NSA groups

No significant differences in demographic data, including age and gender ratio, were observed between the septic arthritis (SA) and non-septic arthritis (NSA) control groups. Table 1 provides a comprehensive summary of the baseline characteristics of the study participants and a detailed overview of the study population.

### Definition of study groups: SA and NSA

Based on positive synovial fluid culture results, 29 children were ultimately diagnosed with septic arthritis (SA). For the 25 patients with negative cultures, a comprehensive evaluation including clinical presentation, laboratory findings, and imaging results led to a final diagnosis of transient synovitis (TS) as the cause of their non-septic arthritis (NSA). The outcomes of all synovial fluid bacterial analyses and lesion locations are presented in the subsequent tables (Table 2).

**Table 2:** Results of all synovial fluid bacterial analyses/ site of lesions.

Pathogenic bacteria	Cases	Percentage	Site of lesions	Cases	Percentage
Staphylococcus aureus	14	44.83%	Hip	9	31.04%
Coagulase-negative Staphylococci (CoNS)^a^	6	20.68%	Knee	9	31.04%
Salmonella	4	13.8%	Shoulder	4	13.79%
Streptococcus agalactiae-(Group B)	3	6.89%	Elbow	2	6.89%
Candida albicans	1	3.45%	Sternoclavicular	2	6.89%
Enterobacter cloacae	1	3.45%	Ankle	3	10.35%
Total	29	100%		29	100%

^a^ CoNS: Coagulase-negative Staphylococci. All CoNS isolates were considered true pathogens based on clinical and laboratory data supporting their pathogenicity ((1) isolation from multiple (≥2) separate blood culture sets; (2) the presence of obvious clinical signs of septic arthritis; and (3) a clear clinical response to targeted anti-staphylococcal therapy).

### Differential diagnostic value of CRP vs. PCT

As shown in Table 3, the median serum CRP level was significantly higher in the SA group [35.05 mg/L (IQR, 22.74–69.99)] compared to the NSA group [4.02 mg/L (IQR, 2.52–5.03); *P* < 0.001]. In contrast, there was no statistically significant difference in median serum PCT levels between the SA group [0.05 ng/ml (IQR, 0.05–0.09)] and the NSA group [0.05 ng/ml (IQR, 0.05–0.25); *P* = 0.324].

**Table 3:** Comparison of PCT and CRP positivity in the SA and NSA groups.

Biomarker	Total (*n* = 54)	Septic arthritis (*n* = 29)	Non-septic arthritis (*n* = 25)	*P* value
CRP, M (Q1, Q3)*	10.495 (4.047, 41.710)	35.050 (22.740, 69.990)	4.020 (2.520, 5.030)	<0.001
PCT, M (Q1, Q3)*	0.050 (0.050, 0.170)	0.050 (0.050, 0.090)	0.050 (0.050, 0.250)	0.324
CRP > 10 mg/L, *n* (%)	30/54 (55.6%)	27/29 (93.1%)	3/25 (12.0%)	<0.001
PCT > 0.25 ng/ml, *n* (%)	6/54 (11.1%)	5/29 (17.2%)	1/25 (4.0%)	0.385

*M, median of dataset; Q1, first quartile of dataset; Q3, third quartile of dataset.

Using the conventional clinical threshold of CRP > 10 mg/L, 27 out of 29 (93.1%) patients in the SA group had elevated levels, compared to only 3 out of 25 (12.0%) in the NSA group (*P* < 0.001). For PCT, using a threshold of >0.25 ng/ml, there was no significant difference in positivity rates between the SA (5/29, 17.2%) and NSA (1/25, 4.0%) groups (*P* = 0.385).

### Comparative diagnostic accuracy of CRP and PCT by ROC analysis

**Figure 1:**
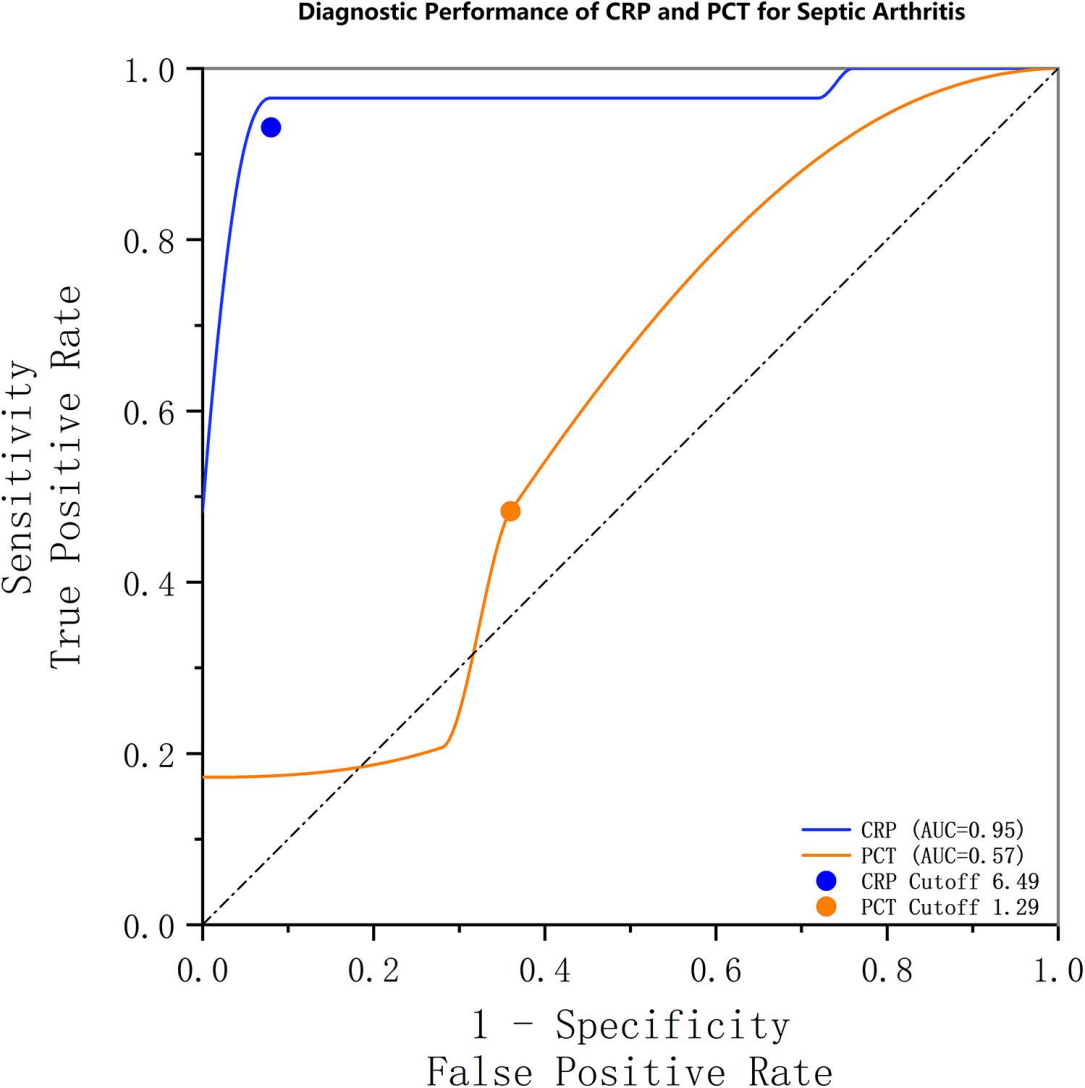
Receiver operating characteristic (ROC) curves demonstrating the diagnostic performance of CRP and PCT for septic arthritis. The area under the curve (AUC) was 0.95 (95% CI, 0.886–0.995) for CRP and 0.57 (95% CI, 0.417–0.731) for PCT. The optimal threshold for CRP was 6.49 mg/L, with a sensitivity and specificity of 96.6% and 92%, respectively. For PCT, the optimal threshold was 1.29 ng/ml, with an associated sensitivity and specificity of 17.2% and 100%, respectively.

The diagnostic performance of CRP and PCT for septic arthritis was evaluated using numerical measurements and the construction of receiver operating characteristic (ROC) curves (Figure 1). The area under the ROC curve (AUC) for CRP was 0.950 (95% CI, 0.886–0.995), corresponding to a Youden's index of 88.6%. The optimal cutoff value for CRP was determined to be 6.49 mg/L, which was associated with a sensitivity of 96.6% and a specificity of 92%. For PCT, the AUC was 0.574 (95% CI, 0.417–0.731), with a Youden's index of 17.2%. The optimal cutoff value for PCT was 1.29 ng/ml, with a sensitivity of 17.2% and specificity of 100%. The detailed findings are presented in Table 4.

**Table 4:** Areas under the ROC curve.

Indicators	AUC	Standard error	*P* value	95% confidence interval	
				Upper limit	Lower limit
CRP (>10 mg/L)	.950	.033	<.001	.886	0.995
PCT (>0.25 ng/ml)	.574	.080	.357	.417	.731

### Sensitivity analysis excluding coagulase-negative staphylococci (CoNS)

To address the potential confounding effect of CoNS and to test the robustness of our primary findings, we performed a sensitivity analysis. We recalculated the diagnostic performance of PCT and CRP after excluding all cases of CoNS infection (*n* = 6) from the SA group. In this refined cohort (*n* = 23), the ROC analysis for PCT yielded an AUC of 0.546, with a newly determined optimal cutoff of 1.025 ng/ml. This cutoff corresponded to a sensitivity of 13% and a specificity of 100%.

**Figure 2:**
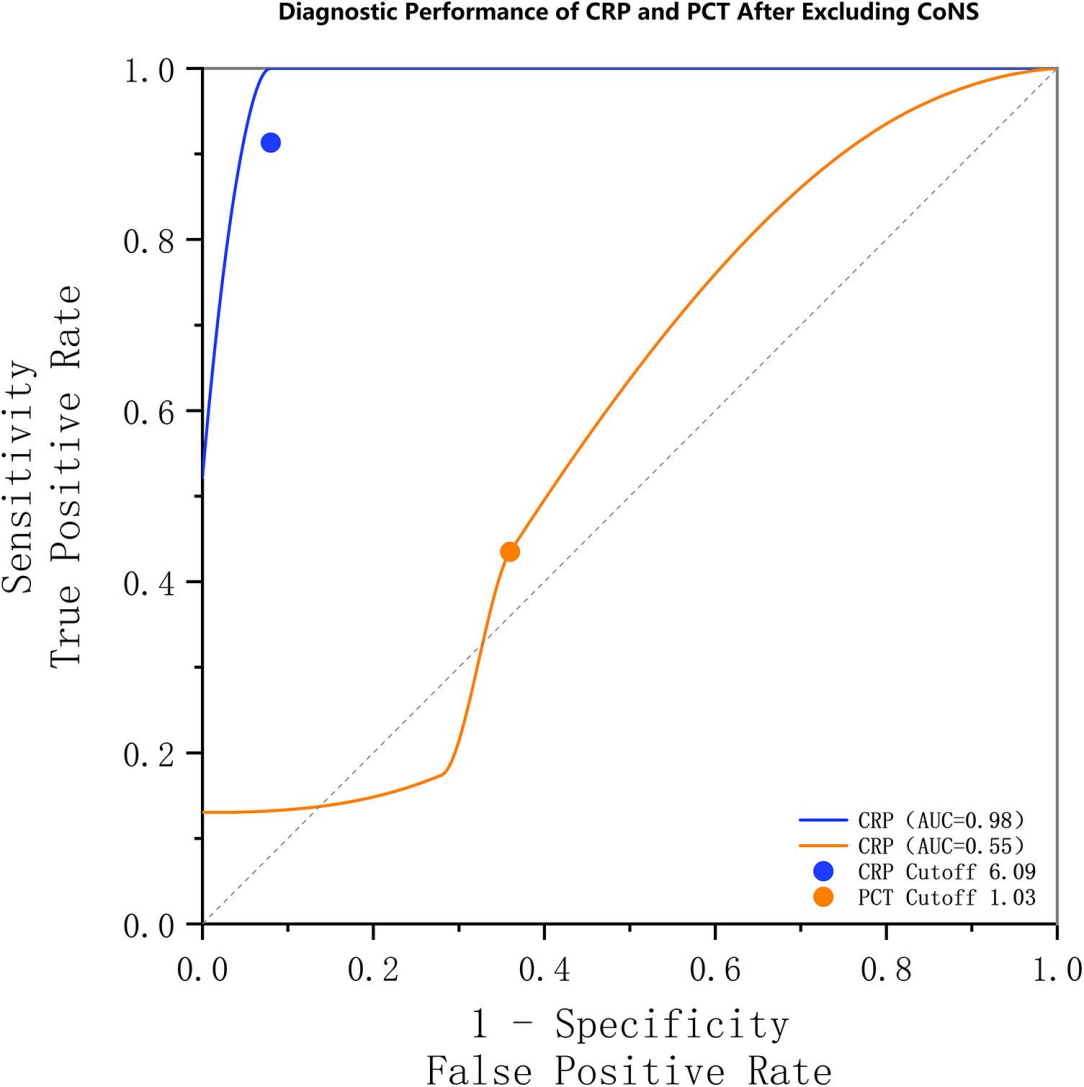
Receiver operating characteristic (ROC) curves from a sensitivity analysis excluding coagulase-negative staphylococci (CoNS). The diagnostic performance of CRP (AUC: 0.98) remained superior to that of PCT (AUC: 0.55). The optimal thresholds, sensitivities, and specificities for CRP and PCT were 6.09 mg/L (100%, 92.0%) and 1.03 ng/ml (13%, 100.0%), respectively.

In contrast, the diagnostic performance of CRP remained exceptionally high in the sensitivity analysis. The ROC analysis for CRP yielded an AUC of 0.975, with an optimal cutoff of 6.085 mg/L, which corresponded to a sensitivity of 100% and a specificity of 92% (Figure 2).

The results of this sensitivity analysis were highly consistent with our primary findings. Notably, the exclusion of CoNS cases, which we had rigorously classified as true pathogens, did not improve the diagnostic utility of PCT; in fact, its AUC slightly decreased. This further solidifies our conclusion that PCT has very limited diagnostic value as a standalone marker for SA in our cohort, whereas CRP demonstrates robust and superior diagnostic performance.

## Discussion

In our study, the median serum CRP level was significantly higher in the SA group [35.05 mg/L (IQR, 22.74–69.99)] compared to the NSA group [4.02 mg/L (IQR, 2.52–5.03); *P* < 0.001]. In contrast, there was no statistically significant difference in median serum PCT levels between the SA group [0.05 ng/ml (IQR, 0.05–0.09)] and the NSA group [0.05 ng/ml (IQR, 0.05–0.25); *P* = 0.324]. Reflecting this profound difference in CRP, we observed a much higher incidence of elevated CRP levels (>10 mg/L) in children with SA than in those without. Among children presenting with acute joint swelling and pain, the serum CRP level demonstrated a sensitivity of 96.6% for detecting SA, with a specificity of 92% and a Youden index of 88.6%. The area under the curve (AUC) was 0.95 (95%CI, 0.886–0.995). This increase in CRP levels in response to SA likely reflects the acute-phase response initiated by the host's immune system to infection. Our findings are consistent with those of Mathews et al., who highlighted the diagnostic potential of CRP. Our previous research demonstrated the significant utility of CRP in differentiating SA from acute synovitis (AS), aligning with the previous research (8, 20). These results further validate the crucial role of CRP in the early detection of septic arthritis in children.

The optimal CRP cutoff of 6.49 mg/L identified in this study is lower than the traditional 10 mg/L threshold, and its significance lies in enhancing diagnostic sensitivity for pediatric septic arthritis (SA). The rationale for this lower cutoff is supported by findings from other researchers, such as Evangelos Spyridakis et al., who reported that in children with SA caused by low-virulence pathogens, the median CRP level was as low as 7 mg/L (21). In our study population, three SA patients presented with CRP levels below 10 mg/L, specifically within the 6–10 mg/L “gray zone,” suggesting that these cases might have been missed under conventional criteria. This underscores that CRP remains an effective biomarker even at modest concentrations in this clinical context. Therefore, for children with high clinical suspicion of SA, a CRP level exceeding 6.5 mg/L should be regarded as a critical red flag, warranting prompt further investigation and consideration of early treatment. This refined cutoff serves as a more sensitive, SA-specific trigger to reduce the risk of diagnostic delay and associated morbidity, thereby complementing—rather than replacing—the general 10 mg/L rule for bacterial infections.

Given the widespread use of procalcitonin (PCT) in diagnosing systemic bacterial infections in recent years, some scholars have subsequently reported its high sensitivity in the diagnosis of septic arthritis [SA, (22–24)]. Shen (25) conducted a meta-analysis of existing literature, demonstrating the potential of serum PCT levels as a diagnostic indicator for bone and joint infection. Bayrak Demirel et al. subsequently also confirmed the potential value of serum PCT in differentiating SA from NSA (26, 27). Fottner reported that serum PCT has high specificity in distinguishing septic arthritis (with a cutoff of PCT > 0.5 ng/ml). However, different scholars have proposed varying cutoff points for SA diagnosis using PCT. Santagada et al. suggested a cutoff value of >0.4 ng/ml.

However, the suitability of PCT as a marker for systemic infection in the early diagnosis of localized suppurative arthritis remains unclear. For instance, Saeed K's (28) research, which encompasses various conditions such as skin infection, diabetic foot infection, infectious arthritis, highlighting the contentious nature of serum PCT's role in the differentiation of infectious and non-infectious arthritis. Streit G (29) conducted a comparative analysis revealing that patients with infectious arthritis had higher peripheral blood PCT levels than those without the condition, although this difference was not statistically significant, while this finding suggests the potential diagnostic value of PCT, it more prominently underscores the necessity for further investigation into its definitive role.

Our study identified three key, yet seemingly contradictory, findings regarding PCT's diagnostic performance: a high optimal cutoff (1.29 ng/ml), a low AUC (0.574), and a non-significant difference in median PCT levels between the SA and NSA groups (*P* = 0.324). These observations are not paradoxical but are, in fact, interconnected statistical manifestations of a single core conclusion: the utility of serum PCT as a standalone diagnostic marker for SA is severely limited in our cohort. This conclusion is further supported by the fact that our derived cutoff is substantially higher than those reported in some other studies [e.g., 0.5 ng/ml by Fottner et al. and 0.4 ng/ml by Santagada et al. (30, 31)], which may reflect differences in cohort characteristics.

The high cutoff of 1.29 ng/ml is best understood as a direct statistical consequence of PCT's poor performance. With an AUC of 0.574, PCT's ability to discriminate between SA and non-SA is minimal, bordering on random chance. In such a scenario, the Youden's index-derived “optimal” cutoff inherently prioritizes maximizing specificity at the severe expense of sensitivity. This is precisely what we observed: a cutoff that achieved 100% specificity but a mere 17.2% sensitivity. Consequently, this high threshold is not a clinically useful decision point but rather a statistical artifact that identifies only a small subset of SA patients with an unusually robust PCT response while missing the majority. This interpretation is directly corroborated by the non-significant intergroup comparison (*P* = 0.324), which provides definitive statistical evidence that PCT, as a continuous variable, failed to distinguish between the SA and NSA groups in our cohort.

A potential concern is that the inclusion of coagulase-negative Staphylococci (CoNS), which are sometimes considered contaminants, might have skewed our results by lowering the overall PCT levels in the SA group. To address this, we performed a sensitivity analysis. Furthermore, a sensitivity analysis excluding all CoNS cases yielded a nearly identical AUC (0.546) and a similarly high, clinically impractical cutoff (1.025 ng/ml), reinforcing that our primary findings are robust and not unduly influenced by the potential inclusion of contaminating organisms.

Having established the statistical robustness of our finding, we now turn to the underlying pathophysiological and clinical reasons for PCT's poor performance in our cohort. Two interrelated factors are paramount. First, the microbiological etiology was a key factor. It is well-established that PCT synthesis is robustly triggered by endotoxins from Gram-negative bacteria (32, 33). In contrast, our cohort was predominantly characterized by Gram-positive infections (e.g., Staphylococcus aureus), which release lipoteichoic acid and other components that are considerably less potent stimulators of PCT production (34, 35). This finding is consistent with a recent study by Niu et al., which demonstrated that PCT levels are significantly higher in Gram-negative bacterial infections compared to Gram-positive infections, making it a powerful biomarker for distinguishing between these two etiologies (36). Second, the clinical stage and extent of infection were equally important. Our study exclusively enrolled patients with culture-confirmed SA, a stricter gold standard that likely captured individuals with early-stage or localized infections who had not yet mounted a significant systemic inflammatory response. Indeed, over half of our cohort (55.17%) presented within 5 days of symptom onset. In such early and localized infections, particularly those caused by less virulent organisms, the inflammatory stimulus is often insufficient to trigger a measurable rise in serum PCT (37). This clinical context not only explains the low sensitivity of PCT but also aligns with the observed PCT-CRP dissociation and underscores the inherent challenges of relying on PCT as a standalone diagnostic marker for SA (38).

In summary, our findings establish a robust evidence chain demonstrating PCT's limited utility in diagnosing SA. The triad of a high cutoff, low AUC, and non-significant intergroup differences confirms that standalone PCT is unreliable for identifying early, localized SA, particularly in Gram-positive-dominated cohorts. Therefore, we caution against over-interpreting or relying solely on PCT for SA diagnosis in this population. Instead, our data reinforce that a comprehensive diagnostic approach, integrating clinical assessment with more reliable biomarkers such as CRP, is crucial for accuracy.

This study has several important limitations. The most significant of which is the relatively small sample size (*n* = 54). A *post-hoc* power analysis revealed that the current study had approximately 58% statistical power to detect a moderate effect size (d = 0.6) at *α* = 0.05, given the sample sizes of 29 in the SA group and 25 in the NSA group. While this cohort is comparable to those in several similar single-center studies, it inherently limits the statistical power of our analysis and increases the risk of a Type II error (failing to detect a true difference). Consequently, the observed non-significant performance of PCT, while compelling, must be interpreted with caution. The small sample size is a direct consequence of the low incidence of pediatric SA in our region, the strict inclusion criteria (e.g., culture-confirmed diagnosis only), and the single-center, retrospective design. Therefore, the generalizability of our findings, particularly the calculated optimal cutoff values and AUCs, may be limited. We strongly advocate for future large-scale, prospective, multicenter studies to validate our conclusions and provide more definitive evidence on the comparative utility of CRP and PCT. A second important limitation is the inherent risk of selection and information bias associated with our single-center, retrospective design. Regarding selection bias, the decision to perform a joint tap—the prerequisite for our gold-standard diagnosis—was at the clinician's discretion. This non-standardized approach likely resulted in the exclusion of patients with milder or atypical symptoms, potentially skewing our cohort towards more severe cases. This effect is compounded by our setting as a tertiary care children's hospital, which further enriches our sample with complex presentations and may limit the generalizability of our findings to primary or community care settings. The single-center nature also inherently restricts the diversity of our population in terms of genetics, socioeconomic factors, and local bacterial epidemiology. Regarding information bias, while we relied on electronic medical records, the key variables of interest—CRP and PCT levels—were measured using objective, standardized laboratory assays, mitigating the risk of measurement error for our primary predictors.

Despite these methodological constraints, the internal validity of our study is strengthened by several factors. First, the use of a culture-confirmed gold standard for diagnosis ensures diagnostic accuracy within our cohort. Second, the objective nature of our primary outcome and predictors minimizes subjective bias in data collection. Most importantly, the congruence of our findings with existing literature, particularly the observed non-significant performance of PCT, reinforces the credibility of our results. While these limitations necessitate cautious interpretation, particularly regarding the optimal cutoff values, they do not invalidate the core conclusion of our study. Instead, they clearly delineate the scope of our findings and provide a robust rationale for future large-scale, prospective, multicenter investigations to achieve broader generalizability.

## Conclusion

Our study demonstrated that CRP is a highly reliable biomarker for diagnosing septic arthritis (SA) in children, outperforming PCT, with an AUC of 0.950 compared to 0.574. At the optimal cutoff of 6.49 mg, CRP achieved 96.6% sensitivity and 92% specificity. These findings strongly support the use of CRP as an effective diagnostic marker for pediatric SA. In conclusion, for pediatric patients with suspected SA, CRP is recommended as a first-line screening marker. CRP > 6.5 mg/L indicates high suspicion of SA, warranting prompt joint aspiration.

However, given the limited sample size, its superiority over procalcitonin (PCT) requires further validation in larger, multicenter, prospective studies. Future research should prioritize: 1. Multicenter prospective validation studies to enhance generalizability; 2. Exploration of age-specific diagnostic cutoff values to improve accuracy across pediatric populations; 3. Development of integrated CRP-PCT diagnostic models to optimize early detection and clinical decision-making.

Such efforts will strengthen diagnostic pathways and ultimately improve outcomes for children with SA. Furthermore, the development of CRP-guided diagnostic pathways could significantly improve early SA detection and management in children, representing an important clinical priority.

## Data Availability

The raw data supporting the conclusions of this article will be made available by the authors, without undue reservation.
